# Data on assessment of groundwater quality with application of ArcGIS in Zanjan, Iran

**DOI:** 10.1016/j.dib.2018.03.059

**Published:** 2018-03-17

**Authors:** Farzaneh Baghal Asghari, Ali Akbar Mohammadi, Mohammad Hadi Dehghani, Mahmood Yousefi

**Affiliations:** a Department of Environmental Health Engineering, School of Public Health, Tehran University of Medical Sciences, Tehran, Iran; b Department of Environmental Health Engineering, Neyshabur University of Medical Sciences, Neyshabur, Iran

**Keywords:** Ground water, Zanjan, Iran

## Abstract

The aim of this study was to Monitoring of physical and chemical characteristics of ground water including Ca^2+^, Mg^2+^, EC, pH, TDS, TH, $HCO_{3}^{-}$ , Na^+^, K^+^, Cl^−^, SAR, %Na and $SO_{4}^{2-}$ in Zanjan city, Iran. For assessing the physic-chemical parameters from 15 wells, water samples 4 times at different times were collected and examined. Data were analyzed using R and Arc GIS software. According to the calculated correlation coefficients, the highest correlation Coefficient belonged to TDS-EC while $HCO_{3}^{-}$ and Cl^−^ showed low and weak correlations. However, Na^+^, Mg^2+^, K^+^, Ca^2+^ exhibited good positive correlations with EC and TDS. The results show that the water in the study area at the time of the study was based on the WHO standards and appropriate for drinking.

**Specifications Table**

**Table:** 

Subject area	Water chemistry
More specific subject area	Describe water subject area
Type of data	Table, Figure
How data was acquired	Analysis for Each sampling point was performed for 4 times at different times that included calcium, magnesium, chloride, temporary and permanent hardness, pH and electrical conductivity (EC). Sulfate analyzed by Hatch spectrophotometer (DR 5000). Total hardness was determined by EDTA method by titration method and TDS was measured gravimetrically.
Data format	Raw, Analyzed
Experimental features	The parameters mentioned in this paper have been analyzed according to Standard Methods for the Examination of Water and Wastewater.
Data source location	Zanjan, Zanja province, Iran
Data accessibility	Data are included in this article and supplement file excel and ArcGIS

**Value of the data**

• Determination of the physical and chemical parameter including Ca^2+^, Mg^2+^, EC, pH, TDS, TH, $HCO_{3}^{-}$, Na^+^, K^+^, Cl^−^, SAR, %Na and $SO_{4}^{2-}$ in ground water was conducted in Zanjan city, Iran.
• Data of this study with Arc GIS can help to better understand the quality of groundwater in this area.
• The results show that the water in the study area at the time of the study was based on the WHO standards and appropriate for drinking.

## 1 Data

**Fig. 1:**
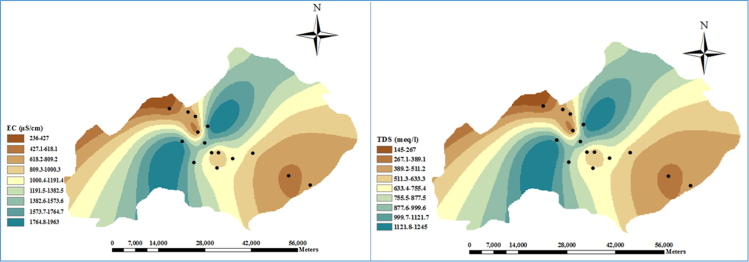
The amount of EC and TDS in the samples studied.

Monitoring of physical and chemical characteristics of ground water including Ca^2+^, Mg^2+^, EC, pH, TDS, TH, $HC{O_{3}}^{-}$ , Na^+^, K^+^, Cl^−^, SAR, %Na and $SO_{4}^{2-}$ was done in Zanjan city, Iran. In this regard data were analyzed using R and Arc GIS software. Table 1 summarizes analysis of the groundwater samples at the study area. Table 2 shows results of Pearson correlation matrix for 10 chemical constituents of the groundwater samples. The TDS and EC level in the study area depicted using the ArcGIS software, as shown in Fig. 1. In this figure, the brighter range represents fewer values, and the darker range is a large value.

**Table 1:** Groundwater quality parameters analyzed in this study.

**Well no**	**UTM**		**EC (μmhos/cm**	**TDS (mg/l)**	**pH**	**meq/l**							**SAR**	**Na%**	**TH**
	**Utmy**	**Utmx**				$HC{O_{3}}^{-}$	**Cl**^**−**^	$SO_{4}^{2-}$	**Ca**^**2+**^	**Mg**^**2+**^	**Na**^**+**^	**K**^**+**^			
P1	4074050	257400	236	145	7.77	1.64	0.25	0.415	1.4	0.44	0.42	0.01	0.44	18.60	92
P 2	4064250	261300	1952	1245	7.47	4.28	4.08	10.44	5.95	6.18	6.80	0.06	2764	35.81	606.35
P 3	4073050	263000	665	425	7.78	2.04	1.24	3.03	3.09	1.04	2.23	0.03	1553	34.88	206.95
P 4	4057850	264850	1568	1005	7.45	3.72	1.93	9.33	4.27	3.75	7.09	0.06	3548.5	46.76	401.15
P 5	4071800	265250	697.5	445	7.74	2.56	0.98	3.11	2.22	0.88	3.64	0.025	2923.5	53.80	154.95
P 6	4067000	265900	518.5	325	7.84	2.56	0.78	1.61	2.87	0.9	1.23	0.02	0.8955	24.51	188.95
P 7	4063750	268100	1747	1100	7.28	2.72	7.52	6.77	7.21	5.96	3.91	0.055	1059.7	19.68	658.5
P 8	4068775	268950	1962	1245	7.3	5.36	4.73	8.8	9.28	4.02	5.73	0.07	2221	30	664.95
P 9	4060825	270050	1039	665	7.73	2.96	1.32	5.61	4.77	1.14	4.07	0.03	2378.5	40.76	295.9
P 10	4056200	271750	1040	665	7.79	3.52	1.67	4.74	3.47	1.21	5.35	0.025	3505.5	53.23	233.95
P 11	4060850	272175	904	570	7.19	3.28	1.74	3.65	3.87	1.57	3.29	0.05	1996	37.5	272
P 12	4059000	276500	1145	715	7.32	4.84	2.87	3.31	5.35	2.19	3.57	0.04	1834.5	31.91	377
P 13	4060600	282475	889	560	7.49	4.28	1.75	2.52	4.29	1.56	2.75	0.03	1641	33.32	292.95
P 14	4053850	293375	442.5	275	7.845	3.04	0.425	0.815	2.44	0.72	1.115	0.015	0.8895	26.045	157.95
P 15	4051050.00	299800	538.33	336.67	7.72	2.93	0.90	1.33	2.40	0.95	1.87	0.02	1453.33	35.77	167.30

**Table 2:** Pearson correlation matrix among the chemical constituents for the groundwater samples.

**Variables**	**K**^**+**^	**Na**^**+**^	**Mg**^**2+**^	**Ca**^**2+**^	$SO_{4}^{2-}$	**Cl**^**−**^	$HCO_{3}^{-}$	**TDS**	**EC**	**TH**
**K**	1.00									
**Na**^**+**^	0.81	1.00								
**Mg**^**2+**^	0.90	0.70	1.00							
**Ca**^**2+**^	0.82	0.62	0.78	1.00						
$SO_{4}^{2-}$	0.90	0.93	0.84	0.75	1.00					
**Cl**	0.78	0.51	0.88	0.86	0.66	1.00				
$HCO_{3}^{-}$	0.60	0.61	0.46	0.71	0.54	0.42	1.00			
**TDS**	0.94	0.87	0.91	0.89	0.95	0.83	0.67	1.00		
**EC**	0.94	0.87	0.91	0.89	0.94	0.83	0.67	0.99	1.00	
**TH**	0.91	0.70	0.93	0.94	0.84	0.92	0.63	0.95	0.96	1.00

## 2 Experimental design, materials and methods

### 2.1 Study area description

**Fig. 2:**
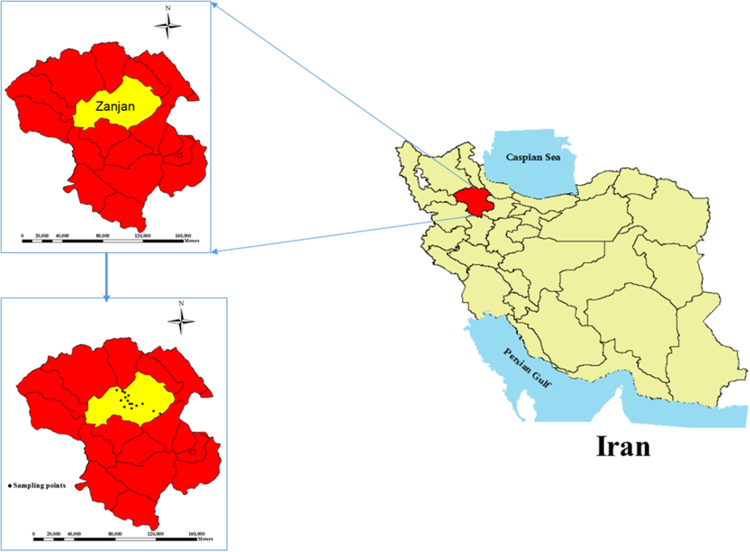
The map and location of sampling points of Zanjan city,Zanjan,Iran.

Zanjan is the capital of Zanjan province in Iran and located about 80 miles south from the Caspian Sea. That coordinates are 36°40′27.6204″N and 48°29′4.0812″E. 15 wells were selected as sampling points. Study area and the sampling points are shown and in Fig. 2.

### 2.2 Materials and methods

For assessing the physicochemical parameters, from 15 wells, water samples 4 times at different times during the year were collected from Zajan city in 2016. Analysis included calcium, magnesium, chloride, temporary and permanent hardness, pH and electrical conductivity (EC) [1], [2], [3], [4], [5]. Sulfate analyzed by Hatch spectrophotometer (DR 5000) [6], [7], [8], [9], [10]. Total hardness was determined by EDTA method by titration method and TDS was measured gravimetrically. All of parameters in this paper have been analyzed according to handbook of Standard Methods for the Examination of Water and Wastewater [1], [2], [3], [4], [5], [6], [7], [8], [9], [10], [11], [12]. Since a simple a method for evaluating the changes of high sodium is the Sodium Adsorption Ratio (SAR) and the sodium percentage (Na %).The excess concentration of sodium in groundwater creates adverse effects as it reacts with the soil and decreases soil permeability and influences plant growth. Sodium percentage is also widely used to evaluate the suitability of water quality for irrigation. The percentage of sodium solution is calculated from the following formula [2] (Table 3).

$$\mathrm{Na}\%=\frac{\mathrm{Na}+K}{\mathrm{Ca}+\mathrm{Mg}+\mathrm{Na}+K}\times 100$$

**Table 3:** sodium percentage (Na%) in present study.

**Parameter**	**Range**	**Water class**
**Na%**	< 20	Excellent
	20–40	Good
	40–60	Permissible
	60–80	Doubtful
	> 80	Unsuitable

## References

[1] Soleimani H., Abbasnia A., Yousefi M., Mohammadi A.A., Changani Khorasgani F. (2018). Data on assessment of groundwater quality for drinking and irrigation in rural area Sarpol-e Zahab city, Kermanshah province, Iran. Data Breif.

[2] Yousefi M., Najafi Saleh H., Mohammad A.A., Mahvi A.H., Ghadrpoori M., Suleimani H. (2017). Data on water quality index for the groundwater in rural area Neyshabur County, Razavi province, Iran. Data Brief..

[3] Yousefi M., Dehghani M.H., Nasab S.M., Taghavimanesh V., Nazmara S., Mohammadi A.A. (2018). Data on trend changes of drinking groundwater resources quality: a case study in Abhar. Data Brief..

[4] Mohammadi A.A., Yousefi M., Mahvi A.H. (2017). Fluoride concentration level in rural area in Poldasht city and daily fluoride intake based on drinking water consumption with temperature. Data Brief..

[5] Abbasnia A., Alimohammadi M., Mahvi A.H., Nabizadeh R., Yousefi M., Mohammadi A.A., Pasalari H., Mirzabeigi M. (2018). Assessment of groundwater quality and evaluation of scaling and corrosiveness potential of drinking water samples in villages of Chabahr city, Sistan and Baluchistan province in Iran. Data Brief..

[6] Asghari F.B., Mohammadi A.A., Aboosaedi Z., Yaseri M., Yousefi M. (2017). Data on fluoride concentration levels in cold and warm season in rural area of Shout (West Azerbaijan, Iran). Data Brief..

[7] Yousefi M., Mohammadi A.A., Yaseri M., Mahvi A.H. (2017). Epidemiology of fluoride and its contribution to fertility, infertility, and abortion: an ecological study in West Azerbaijan Province, Poldasht County, Iran. Fluoride.

[8] Yousefi M., Ghoochani M., Mahvi A.H. (2018). Health risk assessment to fluoride in drinking water of rural residents living in the Poldasht city, Northwest of Iran. Ecotoxicol. Environ. Saf..

[9] Yousefi M., Saleh H.N., Yaseri M., Mahvi A.H., Soleimani H., Saeedi Z. (2018). Data on microbiological quality assessment of rural drinking water supplies in Poldasht county. Data Brief..

[10] Asghari F.B., Jaafari J., Yousefi M., Mohammadi A.A., Dehghanzadeh R. (2018). Evaluation of water corrosion, scaling extent and heterotrophic plate count bacteria in asbestos and polyethylene pipes in drinking water distribution system. Human. Ecol. Risk Assess.: Int. J..

[11] Yousefi M., Saleh H.N., Mahvi A.H., Alimohammadi M., Nabizadeh R., Mohammadi A.A. (2018). Data on corrosion and scaling potential of drinking water resources using stability indices in Jolfa, East Azerbaijan, Iran. Data Brief..

[12] Mohammadi A.A., Yaghmaeian K., Faraji H., Nabizadeh R., Dehghani M.H., Khaili J.K., Mahvi A.H. (2017). Temporal and spatial variation of chemical parameter concentration in drinking water resources of Bandar-e Gaz City using geographic information system. Desalination Water Treat.

